# Supplementary material for: Detailed molecular and epigenetic characterization of the pig IPEC-J2 and chicken SL-29 cell lines
Source: iScience. 2023 Feb 20;26(3):106252. doi: 10.1016/j.isci.2023.106252 (PMC10018572; doi:10.1016/j.isci.2023.106252)
Supplement: Data S1. Complete homer output for identified motifs in Pig IPECJ-2, related to Table 2 — Homer motif analysis results for histone modifications H3K4me1, H3K4me3, H3K27ac, and enhancer elements of pig IPECJ2 cell line. P-values >1e-10 are possible false positives. Within each folder (e.g. peak_files_CTCF) are the html files showing the identified motifs when using homer (e.g. homerResults.html). [file mmc2.zip › S5/Pig_IPECJ_2/peak_files_H3K27ac/knownResults.html]

peak\_files\_H3K27ac - Homer Known Motif Enrichment Results


# Homer Known Motif Enrichment Results (peak\_files\_H3K27ac)

Homer *de novo* Motif Results  
Gene Ontology Enrichment Results  
Known Motif Enrichment Results (txt file)  
Total Target Sequences = 52583, Total Background Sequences = 51852

|  |  |  |  |  |  |  |  |  |  |  |  |
| --- | --- | --- | --- | --- | --- | --- | --- | --- | --- | --- | --- |
| Rank | Motif | Name | P-value | log P-pvalue | q-value (Benjamini) | # Target Sequences with Motif | % of Targets Sequences with Motif | # Background Sequences with Motif | % of Background Sequences with Motif | Motif File | SVG |
| 1 | C G A T C T G A C G T A C A G T A G T C G A T C G A T C A C T G | Pitx1(Homeobox)/Chicken-Pitx1-ChIP-Seq(GSE38910)/Homer | 1e-50 | -1.157e+02 | 0.0000 | 30409.0 | 57.83% | 28306.1 | 54.59% | motif file (matrix) | svg |
| 2 | A T G C A G T C C T G A A G T C C G A T A C G T A G T C A G T C A C G T A T C G G A C T A C G T | Etv2(ETS)/ES-ER71-ChIP-Seq(GSE59402)/Homer | 1e-35 | -8.186e+01 | 0.0000 | 7039.0 | 13.39% | 6015.5 | 11.60% | motif file (matrix) | svg |
| 3 | T C G A T A G C G T C A A C T G A C T G C G T A C G T A C T A G A G C T T C A G | ERG(ETS)/VCaP-ERG-ChIP-Seq(GSE14097)/Homer | 1e-33 | -7.695e+01 | 0.0000 | 12194.0 | 23.19% | 10894.3 | 21.01% | motif file (matrix) | svg |
| 4 | C T G A T A G C T G A C T C A G C T A G G T C A C G T A T C A G A G C T T C A G | ETV4(ETS)/HepG2-ETV4-ChIP-Seq(ENCODE)/Homer | 1e-33 | -7.687e+01 | 0.0000 | 8708.0 | 16.56% | 7601.2 | 14.66% | motif file (matrix) | svg |
| 5 | T C G A A G C T A C G T A C G T A G T C A G T C A C G T A T C G G A C T A T C G | EWS:ERG-fusion(ETS)/CADO\_ES1-EWS:ERG-ChIP-Seq(SRA014231)/Homer | 1e-32 | -7.381e+01 | 0.0000 | 5774.0 | 10.98% | 4891.6 | 9.43% | motif file (matrix) | svg |
| 6 | C T A G T C G A C T G A C G T A T A C G G A C T T C A G T C G A G T C A T G C A T A C G A G C T | IRF2(IRF)/Erythroblas-IRF2-ChIP-Seq(GSE36985)/Homer | 1e-31 | -7.277e+01 | 0.0000 | 973.0 | 1.85% | 643.1 | 1.24% | motif file (matrix) | svg |
| 7 | A C T G C T A G T C G A C G A T C A T G G C T A A T C G C G A T G T A C G C T A A G C T G T A C | Fra1(bZIP)/BT549-Fra1-ChIP-Seq(GSE46166)/Homer | 1e-28 | -6.667e+01 | 0.0000 | 3775.0 | 7.18% | 3101.4 | 5.98% | motif file (matrix) | svg |
| 8 | G A T C T C G A A G T C C G A T C G A T A G T C A T G C A C T G A T C G G A C T | Elk1(ETS)/Hela-Elk1-ChIP-Seq(GSE31477)/Homer | 1e-28 | -6.550e+01 | 0.0000 | 4580.0 | 8.71% | 3839.4 | 7.40% | motif file (matrix) | svg |
| 9 | C T G A A C T G A C T G A G T C A G T C A G C T C T A G T A C G | ZFX(Zf)/mES-Zfx-ChIP-Seq(GSE11431)/Homer | 1e-28 | -6.534e+01 | 0.0000 | 10667.0 | 20.29% | 9531.0 | 18.38% | motif file (matrix) | svg |
| 10 | A C T G C A T G T C G A A C G T A C T G C G T A A T C G A C G T G T A C C G T A G A C T A G T C | Fos(bZIP)/TSC-Fos-ChIP-Seq(GSE110950)/Homer | 1e-28 | -6.529e+01 | 0.0000 | 3950.0 | 7.51% | 3266.8 | 6.30% | motif file (matrix) | svg |
| 11 | C G T A T A G C T A G C T G C A A C T G C T A G C G T A C G T A T C A G G A C T | EHF(ETS)/LoVo-EHF-ChIP-Seq(GSE49402)/Homer | 1e-27 | -6.289e+01 | 0.0000 | 10118.0 | 19.24% | 9029.8 | 17.41% | motif file (matrix) | svg |
| 12 | A G T C C T G A A G T C C G A T C A G T G A T C A T G C A C T G A T C G G A C T | Fli1(ETS)/CD8-FLI-ChIP-Seq(GSE20898)/Homer | 1e-27 | -6.260e+01 | 0.0000 | 8833.0 | 16.80% | 7816.1 | 15.07% | motif file (matrix) | svg |
| 13 | T C G A A C G T C A T G G C T A T A G C C G A T G T A C G C T A A C G T A T G C | AP-1(bZIP)/ThioMac-PU.1-ChIP-Seq(GSE21512)/Homer | 1e-25 | -5.894e+01 | 0.0000 | 5029.0 | 9.56% | 4287.1 | 8.27% | motif file (matrix) | svg |
| 14 | C T A G T C G A G C A T C A T G G C T A T A G C C G A T G T A C C T G A A G C T | JunB(bZIP)/DendriticCells-Junb-ChIP-Seq(GSE36099)/Homer | 1e-25 | -5.891e+01 | 0.0000 | 3599.0 | 6.84% | 2978.8 | 5.74% | motif file (matrix) | svg |
| 15 | C A G T T G C A A C G T A C T G C G T A A T C G C G A T T G A C C G T A A C G T | BATF(bZIP)/Th17-BATF-ChIP-Seq(GSE39756)/Homer | 1e-25 | -5.867e+01 | 0.0000 | 4316.0 | 8.21% | 3633.3 | 7.01% | motif file (matrix) | svg |
| 16 | T C G A C T G A T A G C T G A C T C A G T C A G C G T A C G T A T C A G A G C T | ETV1(ETS)/GIST48-ETV1-ChIP-Seq(GSE22441)/Homer | 1e-24 | -5.729e+01 | 0.0000 | 10859.0 | 20.65% | 9778.6 | 18.86% | motif file (matrix) | svg |
| 17 | T C G A T C G A T A C G G A T C G T C A G T A C C G A T A G C T G T C A T G C A | Nkx3.1(Homeobox)/LNCaP-Nkx3.1-ChIP-Seq(GSE28264)/Homer | 1e-24 | -5.669e+01 | 0.0000 | 15789.0 | 30.03% | 14514.0 | 27.99% | motif file (matrix) | svg |
| 18 | C G T A T G A C T A G C T G C A A C T G A C T G C G T A C G T A T C A G G A C T | ELF3(ETS)/PDAC-ELF3-ChIP-Seq(GSE64557)/Homer | 1e-23 | -5.448e+01 | 0.0000 | 5708.0 | 10.86% | 4943.5 | 9.53% | motif file (matrix) | svg |
| 19 | C A T G C T A G T C G A A C G T A C T G C G T A T A G C C G A T T G A C C G T A A G C T G A T C | Fra2(bZIP)/Striatum-Fra2-ChIP-Seq(GSE43429)/Homer | 1e-23 | -5.443e+01 | 0.0000 | 3187.0 | 6.06% | 2626.6 | 5.07% | motif file (matrix) | svg |
| 20 | T C G A T A G C T G C A A C T G A C T G C G T A C G T A C T A G G A C T T A C G | ETS1(ETS)/Jurkat-ETS1-ChIP-Seq(GSE17954)/Homer | 1e-23 | -5.419e+01 | 0.0000 | 7835.0 | 14.90% | 6937.6 | 13.38% | motif file (matrix) | svg |
| 21 | T A G C T A G C G C A T C A T G A C T G G C T A C G T A A C G T A C T G G A T C | TEAD4(TEA)/Tropoblast-Tead4-ChIP-Seq(GSE37350)/Homer | 1e-23 | -5.365e+01 | 0.0000 | 5931.0 | 11.28% | 5156.7 | 9.94% | motif file (matrix) | svg |
| 22 | C T A G T C G A A C G T A C T G C G T A A T G C A C G T G T A C C G T A A G C T G A T C G T A C | Atf3(bZIP)/GBM-ATF3-ChIP-Seq(GSE33912)/Homer | 1e-22 | -5.241e+01 | 0.0000 | 4522.0 | 8.60% | 3857.0 | 7.44% | motif file (matrix) | svg |
| 23 | G C T A A G T C T A C G T G C A A T C G T C A G G C T A T C G A T C A G A G C T | ELF5(ETS)/T47D-ELF5-ChIP-Seq(GSE30407)/Homer | 1e-21 | -4.998e+01 | 0.0000 | 5728.0 | 10.89% | 4991.5 | 9.63% | motif file (matrix) | svg |
| 24 | C T G A T G C A T A G C T G A C T A C G T C A G C T G A G C T A T C A G G A C T | ELF1(ETS)/Jurkat-ELF1-ChIP-Seq(SRA014231)/Homer | 1e-21 | -4.928e+01 | 0.0000 | 4054.0 | 7.71% | 3444.0 | 6.64% | motif file (matrix) | svg |
| 25 | T A C G T A C G G T A C A T C G T A C G T A C G G T C A C T G A C G T A G A C T | E2F4(E2F)/K562-E2F4-ChIP-Seq(GSE31477)/Homer | 1e-21 | -4.893e+01 | 0.0000 | 3881.0 | 7.38% | 3288.0 | 6.34% | motif file (matrix) | svg |
| 26 | G A T C C T G A A G T C C G A T C G A T G A T C A G T C A C T G A T C G A G C T | Elk4(ETS)/Hela-Elk4-ChIP-Seq(GSE31477)/Homer | 1e-20 | -4.832e+01 | 0.0000 | 4659.0 | 8.86% | 4008.9 | 7.73% | motif file (matrix) | svg |
| 27 | C T G A C T G A C A T G A T C G A G C T A T C G G A C T C A T G C T G A G T C A | Tbr1(T-box)/Cortex-Tbr1-ChIP-Seq(GSE71384)/Homer | 1e-20 | -4.826e+01 | 0.0000 | 9897.0 | 18.82% | 8939.2 | 17.24% | motif file (matrix) | svg |
| 28 | C T G A A T C G A G C T A G C T A C G T T A G C C T G A T A C G C G A T A C G T G A C T A G T C | ISRE(IRF)/ThioMac-LPS-Expression(GSE23622)/Homer | 1e-20 | -4.655e+01 | 0.0000 | 619.0 | 1.18% | 410.7 | 0.79% | motif file (matrix) | svg |
| 29 | T G A C A G T C C T G A T G A C C G T A A C G T A C G T A G T C A G T C C G T A | TEAD1(TEAD)/HepG2-TEAD1-ChIP-Seq(Encode)/Homer | 1e-20 | -4.610e+01 | 0.0000 | 7379.0 | 14.03% | 6569.7 | 12.67% | motif file (matrix) | svg |
| 30 | T C G A T C G A T A G C G T A C T C A G T A C G C G T A C G T A T C A G A G C T | GABPA(ETS)/Jurkat-GABPa-ChIP-Seq(GSE17954)/Homer | 1e-19 | -4.529e+01 | 0.0000 | 6809.0 | 12.95% | 6038.8 | 11.65% | motif file (matrix) | svg |
| 31 | C T A G T A C G A G T C C G T A A G T C A C G T A G T C T C G A C G T A T A C G | Nkx2.1(Homeobox)/LungAC-Nkx2.1-ChIP-Seq(GSE43252)/Homer | 1e-19 | -4.514e+01 | 0.0000 | 18772.0 | 35.70% | 17527.4 | 33.80% | motif file (matrix) | svg |
| 32 | C T A G T C G A C G A T A C T G C G T A T A C G A G C T T G A C G C T A A C G T G A T C T A G C | Fosl2(bZIP)/3T3L1-Fosl2-ChIP-Seq(GSE56872)/Homer | 1e-19 | -4.511e+01 | 0.0000 | 2101.0 | 4.00% | 1691.2 | 3.26% | motif file (matrix) | svg |
| 33 | G A T C G T A C C G A T A C T G A C T G C G T A C G T A A C G T A C T G G A T C | TEAD(TEA)/Fibroblast-PU.1-ChIP-Seq(Unpublished)/Homer | 1e-19 | -4.444e+01 | 0.0000 | 5067.0 | 9.64% | 4412.5 | 8.51% | motif file (matrix) | svg |
| 34 | T G C A C T G A A G T C G T C A A C T G A C T G C G T A C G T A C T G A A G C T | EWS:FLI1-fusion(ETS)/SK\_N\_MC-EWS:FLI1-ChIP-Seq(SRA014231)/Homer | 1e-19 | -4.430e+01 | 0.0000 | 4068.0 | 7.74% | 3486.2 | 6.72% | motif file (matrix) | svg |
| 35 | G C T A G A C T G A C T T G C A C G T A A G T C C G T A T A G C G A T C G A C T | Eomes(T-box)/H9-Eomes-ChIP-Seq(GSE26097)/Homer | 1e-18 | -4.348e+01 | 0.0000 | 15131.0 | 28.77% | 14013.9 | 27.02% | motif file (matrix) | svg |
| 36 | C T G A C T G A T A G C G A T C G C T A G T A C A C G T G A T C T G C A C G T A | Nkx2.5(Homeobox)/HL1-Nkx2.5.biotin-ChIP-Seq(GSE21529)/Homer | 1e-17 | -4.117e+01 | 0.0000 | 14815.0 | 28.17% | 13733.6 | 26.48% | motif file (matrix) | svg |
| 37 | A T G C G A T C C G A T C T A G A C T G G C T A C G T A A G C T A C T G A G C T | TEAD2(TEA)/Py2T-Tead2-ChIP-Seq(GSE55709)/Homer | 1e-17 | -4.094e+01 | 0.0000 | 3618.0 | 6.88% | 3091.8 | 5.96% | motif file (matrix) | svg |
| 38 | C T G A A G T C C G A T A G C T A T G C G T A C A C G T A T C G C A G T G C A T | Elf4(ETS)/BMDM-Elf4-ChIP-Seq(GSE88699)/Homer | 1e-17 | -4.066e+01 | 0.0000 | 7906.0 | 15.03% | 7113.0 | 13.72% | motif file (matrix) | svg |
| 39 | G A C T T C G A C G A T T A C G C T A G T A C G A C T G A T G C G T A C G T A C | Zac1(Zf)/Neuro2A-Plagl1-ChIP-Seq(GSE75942)/Homer | 1e-17 | -4.017e+01 | 0.0000 | 21884.0 | 41.62% | 20625.1 | 39.77% | motif file (matrix) | svg |
| 40 | T A G C C G T A C T G A T A C G C G T A A C G T A C T G A C T G A G T C T A C G C T A G G T A C | YY1(Zf)/Promoter/Homer | 1e-17 | -3.977e+01 | 0.0000 | 571.0 | 1.09% | 385.9 | 0.74% | motif file (matrix) | svg |
| 41 | T C A G T C A G G C T A C G T A T A C G G A C T T C A G T C G A C T G A C G T A T A C G G A C T | IRF8(IRF)/BMDM-IRF8-ChIP-Seq(GSE77884)/Homer | 1e-17 | -3.964e+01 | 0.0000 | 2778.0 | 5.28% | 2328.0 | 4.49% | motif file (matrix) | svg |
| 42 | A G C T C G A T T A C G A T C G A T C G C A G T A G T C A G T C A C T G T A G C | HINFP(Zf)/K562-HINFP.eGFP-ChIP-Seq(Encode)/Homer | 1e-16 | -3.842e+01 | 0.0000 | 3414.0 | 6.49% | 2919.0 | 5.63% | motif file (matrix) | svg |
| 43 | A T G C T C G A A G T C A G C T A C G T G T A C A G T C G C T A C T A G C A T G G T C A C T G A T C A G A G T C | Stat3+il21(Stat)/CD4-Stat3-ChIP-Seq(GSE19198)/Homer | 1e-16 | -3.825e+01 | 0.0000 | 5011.0 | 9.53% | 4403.6 | 8.49% | motif file (matrix) | svg |
| 44 | A G T C G A C T C A G T G T A C A G T C A T C G T C A G A C T G G T C A C G T A | Stat3(Stat)/mES-Stat3-ChIP-Seq(GSE11431)/Homer | 1e-16 | -3.790e+01 | 0.0000 | 3744.0 | 7.12% | 3226.5 | 6.22% | motif file (matrix) | svg |
| 45 | A T G C G A C T A G C T C T A G C G T A C T A G C G A T C T A G A T C G G A T C | Nkx2.2(Homeobox)/NPC-Nkx2.2-ChIP-Seq(GSE61673)/Homer | 1e-16 | -3.772e+01 | 0.0000 | 13000.0 | 24.72% | 12019.2 | 23.18% | motif file (matrix) | svg |
| 46 | G A C T C G A T T C A G G A T C G A C T A G C T A G C T A G T C G A T C C G T A C T A G C T A G T C G A T C G A C T G A | Bcl6(Zf)/Liver-Bcl6-ChIP-Seq(GSE31578)/Homer | 1e-16 | -3.701e+01 | 0.0000 | 10350.0 | 19.68% | 9479.4 | 18.28% | motif file (matrix) | svg |
| 47 | A T C G G A T C G C A T C G T A G T C A A C G T A T G C A G T C | CRX(Homeobox)/Retina-Crx-ChIP-Seq(GSE20012)/Homer | 1e-15 | -3.613e+01 | 0.0000 | 17308.0 | 32.91% | 16211.3 | 31.26% | motif file (matrix) | svg |
| 48 | C T A G T C G A A C G T A C T G C G T A T A G C C G A T G T A C C G T A A G C T G A T C G T A C | Jun-AP1(bZIP)/K562-cJun-ChIP-Seq(GSE31477)/Homer | 1e-15 | -3.565e+01 | 0.0000 | 1463.0 | 2.78% | 1162.2 | 2.24% | motif file (matrix) | svg |
| 49 | C T A G C T A G A G T C T C A G A C T G A C G T A C G T C T G A | MYB(HTH)/ERMYB-Myb-ChIPSeq(GSE22095)/Homer | 1e-15 | -3.545e+01 | 0.0000 | 12381.0 | 23.55% | 11448.9 | 22.08% | motif file (matrix) | svg |
| 50 | T G C A T C G A T A G C G T A C T C A G C T A G G T C A G C T A T C A G G A C T | ETS(ETS)/Promoter/Homer | 1e-15 | -3.537e+01 | 0.0000 | 2378.0 | 4.52% | 1986.0 | 3.83% | motif file (matrix) | svg |
| 51 | C G T A T A C G T C G A A C T G A C T G C G T A C G T A T A C G A G C T T A C G | PU.1(ETS)/ThioMac-PU.1-ChIP-Seq(GSE21512)/Homer | 1e-15 | -3.515e+01 | 0.0000 | 3548.0 | 6.75% | 3062.8 | 5.91% | motif file (matrix) | svg |
| 52 | A G T C G C A T C G T A C G T A G T A C A C G T A C T G G A T C G A T C T C G A | BMYB(HTH)/Hela-BMYB-ChIP-Seq(GSE27030)/Homer | 1e-15 | -3.515e+01 | 0.0000 | 10559.0 | 20.08% | 9699.3 | 18.70% | motif file (matrix) | svg |
| 53 | T G C A C T G A A T G C G T C A A C T G A C T G C G T A C G T A C T A G A G C T | Ets1-distal(ETS)/CD4+-PolII-ChIP-Seq(Barski\_et\_al.)/Homer | 1e-15 | -3.508e+01 | 0.0000 | 2061.0 | 3.92% | 1700.3 | 3.28% | motif file (matrix) | svg |
| 54 | A G C T G A C T C T A G C G T A C A T G C G A T C T A G A T C G G A C T C A G T | Bapx1(Homeobox)/VertebralCol-Bapx1-ChIP-Seq(GSE36672)/Homer | 1e-14 | -3.230e+01 | 0.0000 | 13795.0 | 26.23% | 12851.6 | 24.78% | motif file (matrix) | svg |
| 55 | T C A G C T G A C G T A C G T A T A C G G C A T C T A G C T G A C G T A C G T A T A C G G A C T | IRF1(IRF)/PBMC-IRF1-ChIP-Seq(GSE43036)/Homer | 1e-13 | -3.171e+01 | 0.0000 | 1328.0 | 2.53% | 1058.4 | 2.04% | motif file (matrix) | svg |
| 56 | T C A G A G C T A C G T A C G T G T A C G A T C C G T A C T A G C A T G G T C A C G T A T C G A | STAT4(Stat)/CD4-Stat4-ChIP-Seq(GSE22104)/Homer | 1e-13 | -3.065e+01 | 0.0000 | 7232.0 | 13.75% | 6565.5 | 12.66% | motif file (matrix) | svg |
| 57 | C G T A C G T A C G T A G C A T G C A T A C T G G T A C G A C T C T A G G C T A T A C G G A C T T G A C C G T A A G C T | NFE2L2(bZIP)/HepG2-NFE2L2-ChIP-Seq(Encode)/Homer | 1e-13 | -3.040e+01 | 0.0000 | 491.0 | 0.93% | 340.7 | 0.66% | motif file (matrix) | svg |
| 58 | T G A C C T A G T C A G G T C A C G T A T C A G C G A T T C A G T C G A T G C A C T G A T A G C | PU.1-IRF(ETS:IRF)/Bcell-PU.1-ChIP-Seq(GSE21512)/Homer | 1e-13 | -3.020e+01 | 0.0000 | 10912.0 | 20.75% | 10094.6 | 19.47% | motif file (matrix) | svg |
| 59 | G T C A G C A T A C T G G T A C G A C T A C T G G C T A A T C G C A G T G T A C C G T A A G C T | Nrf2(bZIP)/Lymphoblast-Nrf2-ChIP-Seq(GSE37589)/Homer | 1e-12 | -2.795e+01 | 0.0000 | 394.0 | 0.75% | 266.6 | 0.51% | motif file (matrix) | svg |
| 60 | C G T A C G T A C G T A G C A T G C A T T A C G G T A C G A C T A C T G C G T A A T C G A C G T G T A C C G T A A G C T | Bach1(bZIP)/K562-Bach1-ChIP-Seq(GSE31477)/Homer | 1e-11 | -2.660e+01 | 0.0000 | 438.0 | 0.83% | 305.4 | 0.59% | motif file (matrix) | svg |
| 61 | T G C A A C T G T A C G A T G C A G T C G A C T T C G A A T C G | ZNF711(Zf)/SHSY5Y-ZNF711-ChIP-Seq(GSE20673)/Homer | 1e-10 | -2.491e+01 | 0.0000 | 15943.0 | 30.32% | 15037.6 | 29.00% | motif file (matrix) | svg |
| 62 | A G C T C T A G T G A C C G T A A C G T C G A T A G T C A G T C C T G A C A T G | TEAD3(TEA)/HepG2-TEAD3-ChIP-Seq(Encode)/Homer | 1e-10 | -2.483e+01 | 0.0000 | 8248.0 | 15.69% | 7598.4 | 14.65% | motif file (matrix) | svg |
| 63 | A T G C C T G A A T C G T A C G A G T C C G A T T C A G C G A T C T A G A G C T G T C A G T C A C G T A A G T C C G T A T A C G C T G A | Fox:Ebox(Forkhead,bHLH)/Panc1-Foxa2-ChIP-Seq(GSE47459)/Homer | 1e-10 | -2.361e+01 | 0.0000 | 6261.0 | 11.91% | 5712.8 | 11.02% | motif file (matrix) | svg |
| 64 | C G A T T A C G T G A C G A C T C A T G C G T A T A C G A C G T G T A C C T G A | Bach2(bZIP)/OCILy7-Bach2-ChIP-Seq(GSE44420)/Homer | 1e-10 | -2.347e+01 | 0.0000 | 1247.0 | 2.37% | 1021.9 | 1.97% | motif file (matrix) | svg |
| 65 | A G T C A C G T A C T G A G C T A C G T A C G T G T C A A G T C | Foxo1(Forkhead)/RAW-Foxo1-ChIP-Seq(Fan\_et\_al.)/Homer | 1e-9 | -2.291e+01 | 0.0000 | 13217.0 | 25.14% | 12418.3 | 23.95% | motif file (matrix) | svg |
| 66 | T A G C T C A G C A T G G C A T A G C T C G A T A T G C C G T A C G T A G T C A | CHR(?)/Hela-CellCycle-Expression/Homer | 1e-9 | -2.274e+01 | 0.0000 | 5293.0 | 10.07% | 4800.2 | 9.26% | motif file (matrix) | svg |
| 67 | C G A T C T A G A C G T G T C A C G T A C G T A A G T C C G T A | Foxo3(Forkhead)/U2OS-Foxo3-ChIP-Seq(E-MTAB-2701)/Homer | 1e-9 | -2.245e+01 | 0.0000 | 5253.0 | 9.99% | 4765.5 | 9.19% | motif file (matrix) | svg |
| 68 | T A C G T C G A C A G T A C T G G C T A A T G C C G A T G T A C C G T A A C T G T A G C C G T A | NF-E2(bZIP)/K562-NFE2-ChIP-Seq(GSE31477)/Homer | 1e-9 | -2.240e+01 | 0.0000 | 415.0 | 0.79% | 296.2 | 0.57% | motif file (matrix) | svg |
| 69 | C T A G C T A G C G T A C G T A T A C G C G A T C T A G C T G A C T G A C G T A T A C G G A C T | PU.1:IRF8(ETS:IRF)/pDC-Irf8-ChIP-Seq(GSE66899)/Homer | 1e-9 | -2.226e+01 | 0.0000 | 1532.0 | 2.91% | 1285.0 | 2.48% | motif file (matrix) | svg |
| 70 | C T G A T A C G G C A T A G C T A G C T A G T C T C G A A C T G C A G T A G C T A G C T G A T C | IRF3(IRF)/BMDM-Irf3-ChIP-Seq(GSE67343)/Homer | 1e-9 | -2.207e+01 | 0.0000 | 2886.0 | 5.49% | 2537.7 | 4.89% | motif file (matrix) | svg |
| 71 | A G C T T G A C C G A T C G A T C T A G A C G T C A G T C A G T G C T A A G T C | FOXK1(Forkhead)/HEK293-FOXK1-ChIP-Seq(GSE51673)/Homer | 1e-9 | -2.186e+01 | 0.0000 | 7480.0 | 14.22% | 6897.9 | 13.30% | motif file (matrix) | svg |
| 72 | T A G C G A T C G T A C C T G A G C A T C T G A C G T A T G C A C G T A G A T C | Hoxa13(Homeobox)/ChickenMSG-Hoxa13.Flag-ChIP-Seq(GSE86088)/Homer | 1e-9 | -2.165e+01 | 0.0000 | 19807.0 | 37.67% | 18862.3 | 36.37% | motif file (matrix) | svg |
| 73 | C G A T A C G T A C G T A C G T C G T A A G C T C A G T C T A G A T C G A C T G | HOXB13(Homeobox)/ProstateTumor-HOXB13-ChIP-Seq(GSE56288)/Homer | 1e-9 | -2.141e+01 | 0.0000 | 8677.0 | 16.50% | 8052.6 | 15.53% | motif file (matrix) | svg |
| 74 | T A G C G T A C C G T A C T A G A C T G T G C A C G T A A T G C C G T A A T C G | AR-halfsite(NR)/LNCaP-AR-ChIP-Seq(GSE27824)/Homer | 1e-9 | -2.139e+01 | 0.0000 | 25109.0 | 47.75% | 24072.7 | 46.42% | motif file (matrix) | svg |
| 75 | T A G C C G A T T A C G A C T G A G T C A C T G A T C G A T C G C G T A C T G A | E2F1(E2F)/Hela-E2F1-ChIP-Seq(GSE22478)/Homer | 1e-9 | -2.103e+01 | 0.0000 | 2178.0 | 4.14% | 1886.7 | 3.64% | motif file (matrix) | svg |
| 76 | G A C T C T A G C T A G A G T C T G C A A C T G A C G T A C G T C T A G T C A G | AMYB(HTH)/Testes-AMYB-ChIP-Seq(GSE44588)/Homer | 1e-9 | -2.095e+01 | 0.0000 | 10409.0 | 19.79% | 9728.6 | 18.76% | motif file (matrix) | svg |
| 77 | A C T G A C G T C A T G A T C G A T C G T G A C A C T G A T C G A T C G T G C A C T G A C G T A | E2F3(E2F)/MEF-E2F3-ChIP-Seq(GSE71376)/Homer | 1e-9 | -2.089e+01 | 0.0000 | 5564.0 | 10.58% | 5077.3 | 9.79% | motif file (matrix) | svg |
| 78 | A T C G A G T C A G T C G A C T A T G C C T G A C T A G A C T G T A C G G T A C C T G A C G A T | AP-2gamma(AP2)/MCF7-TFAP2C-ChIP-Seq(GSE21234)/Homer | 1e-8 | -2.040e+01 | 0.0000 | 10495.0 | 19.96% | 9819.7 | 18.94% | motif file (matrix) | svg |
| 79 | T C G A T G A C G C A T A G C T C A G T G A T C G C T A G A T C G A C T A C G T G C A T A G T C | PRDM1(Zf)/Hela-PRDM1-ChIP-Seq(GSE31477)/Homer | 1e-8 | -1.993e+01 | 0.0000 | 4511.0 | 8.58% | 4086.2 | 7.88% | motif file (matrix) | svg |
| 80 | A C G T G A C T A T G C G C T A C T G A C T A G A C T G G A C T A G T C C G T A | Nr5a2(NR)/mES-Nr5a2-ChIP-Seq(GSE19019)/Homer | 1e-8 | -1.968e+01 | 0.0000 | 3971.0 | 7.55% | 3577.8 | 6.90% | motif file (matrix) | svg |
| 81 | C T G A C T A G A C T G G C A T C T A G G C A T A T C G C T G A C G T A G T C A | Tbx21(T-box)/GM12878-TBX21-ChIP-Seq(Encode)/Homer | 1e-8 | -1.893e+01 | 0.0000 | 6848.0 | 13.02% | 6327.8 | 12.20% | motif file (matrix) | svg |
| 82 | A T G C C T G A G A C T A C G T A C G T G T A C G A T C C G A T C T A G C A T G C G T A C G T A C T G A G A C T | STAT1(Stat)/HelaS3-STAT1-ChIP-Seq(GSE12782)/Homer | 1e-8 | -1.893e+01 | 0.0000 | 2256.0 | 4.29% | 1973.6 | 3.81% | motif file (matrix) | svg |
| 83 | A G C T A G T C A G T C A C G T C T A G A C G T A C G T A C G T C G T A A G T C G A T C C G T A | FOXP1(Forkhead)/H9-FOXP1-ChIP-Seq(GSE31006)/Homer | 1e-8 | -1.878e+01 | 0.0000 | 3081.0 | 5.86% | 2746.1 | 5.30% | motif file (matrix) | svg |
| 84 | C G A T T A C G T G C A G T A C G A T C G A C T A G C T A C G T A T C G G T A C G A T C G T A C G A T C G T C A | PPARE(NR),DR1/3T3L1-Pparg-ChIP-Seq(GSE13511)/Homer | 1e-7 | -1.773e+01 | 0.0000 | 7753.0 | 14.74% | 7212.1 | 13.91% | motif file (matrix) | svg |
| 85 | T C G A C A T G C A T G T A C G G T A C T C G A A G T C C A G T C T G A C G T A A G T C G A C T | ZNF264(Zf)/HEK293-ZNF264.GFP-ChIP-Seq(GSE58341)/Homer | 1e-7 | -1.686e+01 | 0.0000 | 4449.0 | 8.46% | 4059.5 | 7.83% | motif file (matrix) | svg |
| 86 | T G A C C T G A C T A G T C G A C T G A A T G C C G T A A C T G G C A T G T A C G C A T A T C G G C A T A G C T G A T C | PR(NR)/T47D-PR-ChIP-Seq(GSE31130)/Homer | 1e-7 | -1.682e+01 | 0.0000 | 14059.0 | 26.74% | 13335.4 | 25.72% | motif file (matrix) | svg |
| 87 | C A G T A C T G T C A G T G C A G C T A A T G C T C G A A T C G G T C A T G C A | ZNF189(Zf)/HEK293-ZNF189.GFP-ChIP-Seq(GSE58341)/Homer | 1e-7 | -1.632e+01 | 0.0000 | 6026.0 | 11.46% | 5572.5 | 10.75% | motif file (matrix) | svg |
| 88 | C G T A C T A G C A T G A G C T A C T G C G A T A T C G C G T A G T C A G T C A | Tbet(T-box)/CD8-Tbet-ChIP-Seq(GSE33802)/Homer | 1e-7 | -1.616e+01 | 0.0000 | 7190.0 | 13.67% | 6692.1 | 12.91% | motif file (matrix) | svg |
| 89 | T G C A A T G C A C G T A C G T A C G T A T G C C T A G A C G T A C G T A G C T G A T C A G C T | T1ISRE(IRF)/ThioMac-Ifnb-Expression/Homer | 1e-6 | -1.555e+01 | 0.0000 | 109.0 | 0.21% | 63.4 | 0.12% | motif file (matrix) | svg |
| 90 | A T G C G A C T A C G T C T A G A C G T A C G T A C G T C T G A G A T C G C T A A G C T C G T A | Foxa2(Forkhead)/Liver-Foxa2-ChIP-Seq(GSE25694)/Homer | 1e-6 | -1.540e+01 | 0.0000 | 5471.0 | 10.40% | 5052.3 | 9.74% | motif file (matrix) | svg |
| 91 | G T A C G C T A T C A G C T G A C T A G C A T G A G C T G A T C T G C A T C G A C T G A A C T G C A G T A G T C G A T C G C T A | HNF4a(NR),DR1/HepG2-HNF4a-ChIP-Seq(GSE25021)/Homer | 1e-6 | -1.535e+01 | 0.0000 | 3396.0 | 6.46% | 3075.3 | 5.93% | motif file (matrix) | svg |
| 92 | T A G C G C T A A C T G C G T A A C G T C G T A C G T A T A C G T C A G T C G A | Gata1(Zf)/K562-GATA1-ChIP-Seq(GSE18829)/Homer | 1e-6 | -1.523e+01 | 0.0000 | 4117.0 | 7.83% | 3761.9 | 7.25% | motif file (matrix) | svg |
| 93 | T A G C C G A T A C G T A G C T A G C T A G T C A T G C A G T C A C T G A T G C A T G C G C T A | E2F7(E2F)/Hela-E2F7-ChIP-Seq(GSE32673)/Homer | 1e-6 | -1.497e+01 | 0.0000 | 986.0 | 1.88% | 828.0 | 1.60% | motif file (matrix) | svg |
| 94 | C T G A C T G A C T G A A T G C G A T C C A T G A C T G G A C T G A C T G C A T C G T A C G T A A G T C G T A C C T G A A T C G G C A T G A C T G A C T A G C T | GRHL2(CP2)/HBE-GRHL2-ChIP-Seq(GSE46194)/Homer | 1e-6 | -1.487e+01 | 0.0000 | 2327.0 | 4.43% | 2071.5 | 3.99% | motif file (matrix) | svg |
| 95 | T G A C T C G A C T G A C T G A A T G C G A T C C T A G T A C G G A C T G A C T G A T C T C G A C T G A C T G A A T G C G A T C C T A G A T C G G A C T G A C T | Tcfcp2l1(CP2)/mES-Tcfcp2l1-ChIP-Seq(GSE11431)/Homer | 1e-6 | -1.465e+01 | 0.0000 | 895.0 | 1.70% | 746.1 | 1.44% | motif file (matrix) | svg |
| 96 | A G T C A G T C C G A T A C G T A C G T A C T G A C G T A G C T A G T C A G T C | Sox4(HMG)/proB-Sox4-ChIP-Seq(GSE50066)/Homer | 1e-6 | -1.444e+01 | 0.0000 | 5872.0 | 11.17% | 5449.2 | 10.51% | motif file (matrix) | svg |
| 97 | T C A G C T A G C T A G T G C A C G A T C G A T C G T A C T A G | GSC(Homeobox)/FrogEmbryos-GSC-ChIP-Seq(DRA000576)/Homer | 1e-6 | -1.410e+01 | 0.0000 | 8946.0 | 17.01% | 8418.3 | 16.23% | motif file (matrix) | svg |
| 98 | C T A G T A G C A G T C G T C A C T G A A C G T C G T A C G T A C G T A G C T A | Hoxd13(Homeobox)/ChickenMSG-Hoxd13.Flag-ChIP-Seq(GSE86088)/Homer | 1e-6 | -1.383e+01 | 0.0000 | 13205.0 | 25.11% | 12559.7 | 24.22% | motif file (matrix) | svg |
| 99 | A T G C A G T C G C T A C G A T C G T A G C A T G C T A C G A T C T A G C A T G T G A C G T C A | CArG(MADS)/PUER-Srf-ChIP-Seq(Sullivan\_et\_al.)/Homer | 1e-5 | -1.330e+01 | 0.0000 | 2357.0 | 4.48% | 2113.2 | 4.08% | motif file (matrix) | svg |
| 100 | T C A G C T G A C T A G C A T G A C G T A T G C C T G A C T G A C T G A C T A G C A T G A C G T A T G C C T G A | TR4(NR),DR1/Hela-TR4-ChIP-Seq(GSE24685)/Homer | 1e-5 | -1.299e+01 | 0.0000 | 893.0 | 1.70% | 753.6 | 1.45% | motif file (matrix) | svg |
| 101 | G C T A A T C G G C T A G A C T G C T A T C G A T A G C T C G A | GATA3(Zf)/iTreg-Gata3-ChIP-Seq(GSE20898)/Homer | 1e-5 | -1.252e+01 | 0.0000 | 10850.0 | 20.63% | 10293.5 | 19.85% | motif file (matrix) | svg |
| 102 | A C G T G A C T T A G C C G T A C T G A C A T G C T A G G A C T G A T C C G T A | Nr5a2(NR)/Pancreas-LRH1-ChIP-Seq(GSE34295)/Homer | 1e-5 | -1.248e+01 | 0.0000 | 5195.0 | 9.88% | 4826.5 | 9.31% | motif file (matrix) | svg |
| 103 | C T A G C G T A G T C A C G T A A G T C G A T C A G C T C T A G C G T A A C G T G T C A G A T C | Six2(Homeobox)/NephronProgenitor-Six2-ChIP-Seq(GSE39837)/Homer | 1e-5 | -1.247e+01 | 0.0000 | 7201.0 | 13.69% | 6758.2 | 13.03% | motif file (matrix) | svg |
| 104 | C G T A C G T A C G T A A G T C A G C T C T G A A C T G A C T G A G C T A G T C C G T A C T A G C T A G C T A G T G C A | RORa(NR)/Liver-Rora-ChIP-Seq(GSE101115)/Homer | 1e-5 | -1.211e+01 | 0.0000 | 1039.0 | 1.98% | 892.6 | 1.72% | motif file (matrix) | svg |
| 105 | A G C T A G C T T A G C A T C G A G T C A C T G A T G C A T C G T C G A C T G A T C G A C T G A | E2F(E2F)/Hela-CellCycle-Expression/Homer | 1e-5 | -1.182e+01 | 0.0000 | 417.0 | 0.79% | 330.5 | 0.64% | motif file (matrix) | svg |
| 106 | A G C T A G T C A T G C A G C T A C G T C G T A A C G T A G T C C G A T A T G C | Gata2(Zf)/K562-GATA2-ChIP-Seq(GSE18829)/Homer | 1e-5 | -1.156e+01 | 0.0000 | 4718.0 | 8.97% | 4381.6 | 8.45% | motif file (matrix) | svg |
| 107 | A C T G C A T G G C T A T C G A G C T A A G C T A G C T G T A C A G T C T G A C | NFkB-p65-Rel(RHD)/ThioMac-LPS-Expression(GSE23622)/Homer | 1e-4 | -1.095e+01 | 0.0001 | 626.0 | 1.19% | 521.9 | 1.01% | motif file (matrix) | svg |
| 108 | G C T A T A G C A G C T A T C G G T C A C G T A G C T A A T G C G A T C C T G A | IRF4(IRF)/GM12878-IRF4-ChIP-Seq(GSE32465)/Homer | 1e-4 | -1.093e+01 | 0.0001 | 3176.0 | 6.04% | 2914.8 | 5.62% | motif file (matrix) | svg |
| 109 | T G C A G C A T A G C T G C A T A G T C A G T C A G T C C T G A A C T G T C G A T C G A C A G T A T C G A G T C G A T C | ZNF143\|STAF(Zf)/CUTLL-ZNF143-ChIP-Seq(GSE29600)/Homer | 1e-4 | -1.074e+01 | 0.0001 | 2128.0 | 4.05% | 1921.5 | 3.71% | motif file (matrix) | svg |
| 110 | C T G A A T G C C G T A A C G T A G T C A G T C A C G T A C T G A T C G G C A T | SPDEF(ETS)/VCaP-SPDEF-ChIP-Seq(SRA014231)/Homer | 1e-4 | -1.066e+01 | 0.0001 | 8360.0 | 15.90% | 7911.7 | 15.26% | motif file (matrix) | svg |
| 111 | A G T C G A C T C A G T A C T G C T A G T G A C G C T A A T G C G C A T A T C G C G A T A C T G G A T C G T A C G T C A C T G A | NF1(CTF)/LNCAP-NF1-ChIP-Seq(Unpublished)/Homer | 1e-4 | -1.063e+01 | 0.0001 | 2849.0 | 5.42% | 2606.7 | 5.03% | motif file (matrix) | svg |
| 112 | T A C G A T G C G C T A A C T G C G T A A C G T C G T A C T G A T A C G T C G A | Gata4(Zf)/Heart-Gata4-ChIP-Seq(GSE35151)/Homer | 1e-4 | -1.015e+01 | 0.0002 | 7313.0 | 13.91% | 6906.0 | 13.32% | motif file (matrix) | svg |
| 113 | C G T A C T G A C T A G C T G A A G T C G C T A C G A T A T C G G A C T G A T C A G T C C T G A C T A G C T A G A G T C G C T A C G A T C T A G G A T C G A T C | p73(p53)/Trachea-p73-ChIP-Seq(PRJNA310161)/Homer | 1e-4 | -1.010e+01 | 0.0002 | 235.0 | 0.45% | 177.4 | 0.34% | motif file (matrix) | svg |
| 114 | T G C A C T A G C T A G C T G A C A T G A C T G T G C A G A T C G T C A T G C A G T C A G T C A A G C T C T A G G C A T | ZNF675(Zf)/HEK293-ZNF675.GFP-ChIP-Seq(GSE58341)/Homer | 1e-4 | -9.922e+00 | 0.0002 | 1004.0 | 1.91% | 874.4 | 1.69% | motif file (matrix) | svg |
| 115 | T G A C C G A T C T G A C T A G C T A G A C G T A T G C T G C A T C G A C T G A C T A G C A T G A C G T A G T C C G T A | PPARa(NR),DR1/Liver-Ppara-ChIP-Seq(GSE47954)/Homer | 1e-4 | -9.777e+00 | 0.0002 | 9194.0 | 17.48% | 8738.4 | 16.85% | motif file (matrix) | svg |
| 116 | T A C G T A G C G C T A C G A T C T A G A C G T C A G T C A G T G C T A A G T C G T C A G C A T | FOXK2(Forkhead)/U2OS-FOXK2-ChIP-Seq(E-MTAB-2204)/Homer | 1e-4 | -9.689e+00 | 0.0002 | 4334.0 | 8.24% | 4039.6 | 7.79% | motif file (matrix) | svg |
| 117 | G C T A C G T A A G T C A C G T T C G A T A C G A C T G A G C T A G T C T C G A | RORgt(NR)/EL4-RORgt.Flag-ChIP-Seq(GSE56019)/Homer | 1e-4 | -9.520e+00 | 0.0003 | 919.0 | 1.75% | 798.9 | 1.54% | motif file (matrix) | svg |
| 118 | G C T A C G T A A G T C A C G T T C G A T A C G A C T G A G C T A G T C T C G A | RORgt(NR)/EL4-RORgt.Flag-ChIP-Seq(GSE56019)/Homer | 1e-4 | -9.520e+00 | 0.0003 | 919.0 | 1.75% | 798.9 | 1.54% | motif file (matrix) | svg |
| 119 | C G T A A C T G C G T A A C G T C G T A C T G A T C A G T G C A A G C T T G A C | TRPS1(Zf)/MCF7-TRPS1-ChIP-Seq(GSE107013)/Homer | 1e-4 | -9.486e+00 | 0.0003 | 14643.0 | 27.85% | 14058.6 | 27.11% | motif file (matrix) | svg |
| 120 | T G C A T A G C G A C T T G C A T G A C T G C A C G T A A G C T A G C T A G T C A G T C G T A C | GFY(?)/Promoter/Homer | 1e-4 | -9.462e+00 | 0.0003 | 376.0 | 0.72% | 303.7 | 0.59% | motif file (matrix) | svg |
| 121 | C T G A C T A G A T C G A G C T A C T G G A C T A G T C C T G A | Tbx5(T-box)/HL1-Tbx5.biotin-ChIP-Seq(GSE21529)/Homer | 1e-4 | -9.393e+00 | 0.0003 | 21571.0 | 41.02% | 20854.9 | 40.22% | motif file (matrix) | svg |
| 122 | A G C T C T G A C T A G C T A G A C T G T A G C T G C A T C G A C T G A C T A G C A T G A C G T A T G C T C G A | RXR(NR),DR1/3T3L1-RXR-ChIP-Seq(GSE13511)/Homer | 1e-3 | -9.085e+00 | 0.0004 | 9469.0 | 18.01% | 9021.0 | 17.40% | motif file (matrix) | svg |
| 123 | T A C G C T G A T C G A C G A T C T A G C T A G T C G A C T G A T C G A T C G A C G T A T C G A G C A T C A T G C G T A T A C G G C A T T G A C C G T A A G C T | NFAT:AP1(RHD,bZIP)/Jurkat-NFATC1-ChIP-Seq(Jolma\_et\_al.)/Homer | 1e-3 | -8.922e+00 | 0.0005 | 1313.0 | 2.50% | 1170.8 | 2.26% | motif file (matrix) | svg |
| 124 | A G C T A C G T A C T G A T G C A G T C C G T A C T G A T A C G | NF1-halfsite(CTF)/LNCaP-NF1-ChIP-Seq(Unpublished)/Homer | 1e-3 | -8.887e+00 | 0.0005 | 12746.0 | 24.24% | 12219.6 | 23.56% | motif file (matrix) | svg |
| 125 | C A T G A G T C A G C T C G T A C G A T C G A T G C A T G C A T C G A T C T G A C A T G T G A C | Mef2d(MADS)/Retina-Mef2d-ChIP-Seq(GSE61391)/Homer | 1e-3 | -8.845e+00 | 0.0005 | 1491.0 | 2.84% | 1338.2 | 2.58% | motif file (matrix) | svg |
| 126 | C T G A T A G C G C T A C G A T A T C G A G T C G A T C G A T C C T A G T C A G T C A G G T A C G C T A C A G T | p53(p53)/mES-cMyc-ChIP-Seq(GSE11431)/Homer | 1e-3 | -8.810e+00 | 0.0005 | 110.0 | 0.21% | 75.8 | 0.15% | motif file (matrix) | svg |
| 127 | G T C A T C G A C T A G C T A G A G T C G T C A C G A T C T A G G A C T G A T C G A T C T C A G C T A G C T G A A G T C G C T A C A G T T C A G G A T C G A T C | p63(p53)/Keratinocyte-p63-ChIP-Seq(GSE17611)/Homer | 1e-3 | -8.791e+00 | 0.0005 | 1823.0 | 3.47% | 1652.9 | 3.19% | motif file (matrix) | svg |
| 128 | T C G A T G C A A C T G G T C A A G T C A G T C A G T C A G T C A G C T T G A C | LRF(Zf)/Erythroblasts-ZBTB7A-ChIP-Seq(GSE74977)/Homer | 1e-3 | -8.489e+00 | 0.0007 | 13578.0 | 25.82% | 13042.6 | 25.15% | motif file (matrix) | svg |
| 129 | A C G T C T A G C G T A A G T C G T A C A C G T A C G T A C G T G T C A G T A C T G A C G A C T | Nur77(NR)/K562-NR4A1-ChIP-Seq(GSE31363)/Homer | 1e-3 | -8.259e+00 | 0.0009 | 1226.0 | 2.33% | 1094.9 | 2.11% | motif file (matrix) | svg |
| 130 | A T C G T C G A G A C T A T C G T G A C A C G T C T A G A C T G C G T A A C T G A G T C G T A C | ZNF415(Zf)/HEK293-ZNF415.GFP-ChIP-Seq(GSE58341)/Homer | 1e-3 | -8.096e+00 | 0.0010 | 3896.0 | 7.41% | 3642.5 | 7.02% | motif file (matrix) | svg |
| 131 | T C G A C G T A A G T C A G C T C G T A A G T C T C G A G C T A G A C T C G A T A G T C A G T C A G T C C T G A T C A G T G C A T C G A C A G T A T C G A G T C | GFY-Staf(?,Zf)/Promoter/Homer | 1e-3 | -8.078e+00 | 0.0010 | 248.0 | 0.47% | 195.8 | 0.38% | motif file (matrix) | svg |
| 132 | A G T C C G A T A C T G A T C G T G A C G C T A C A T G A T C G T G A C C G A T A C T G T A G C G T A C G T C A | Tlx?(NR)/NPC-H3K4me1-ChIP-Seq(GSE16256)/Homer | 1e-3 | -7.896e+00 | 0.0012 | 2707.0 | 5.15% | 2504.8 | 4.83% | motif file (matrix) | svg |
| 133 | A G C T A T G C G A C T G C A T C G T A A G C T G T A C C G A T A T C G A G T C | Gata6(Zf)/HUG1N-GATA6-ChIP-Seq(GSE51936)/Homer | 1e-3 | -7.657e+00 | 0.0016 | 6709.0 | 12.76% | 6369.9 | 12.28% | motif file (matrix) | svg |
| 134 | A G T C C G A T C T G A C G T A A C G T C A G T T C A G T G A C | Isl1(Homeobox)/Neuron-Isl1-ChIP-Seq(GSE31456)/Homer | 1e-3 | -7.310e+00 | 0.0022 | 14082.0 | 26.78% | 13567.6 | 26.16% | motif file (matrix) | svg |
| 135 | T C A G C G T A A G T C A G C T C G T A A G T C C T G A C G T A A G T C G C A T A G T C A G T C A G T C C T G A A C T G T G C A T C G A C A T G A T C G G A T C | Ronin(THAP)/ES-Thap11-ChIP-Seq(GSE51522)/Homer | 1e-3 | -7.156e+00 | 0.0025 | 134.0 | 0.25% | 99.2 | 0.19% | motif file (matrix) | svg |
| 136 | G C T A C G A T C A T G A T G C A G T C A G T C G A C T T A C G T C G A C T A G A C T G T A G C | AP-2alpha(AP2)/Hela-AP2alpha-ChIP-Seq(GSE31477)/Homer | 1e-3 | -7.149e+00 | 0.0025 | 8201.0 | 15.60% | 7830.9 | 15.10% | motif file (matrix) | svg |
| 137 | T G C A A G C T A C G T C T A G G A T C C T A G G A T C G T C A C T G A A G T C | CEBP(bZIP)/ThioMac-CEBPb-ChIP-Seq(GSE21512)/Homer | 1e-3 | -6.996e+00 | 0.0029 | 4291.0 | 8.16% | 4041.6 | 7.79% | motif file (matrix) | svg |
| 138 | A G T C G A T C A G T C C G T A A T C G C A G T A G T C G T A C C T G A A C T G T C A G A G C T A G C T A G C T A G C T | PRDM15(Zf)/ESC-Prdm15-ChIP-Seq(GSE73694)/Homer | 1e-3 | -6.940e+00 | 0.0031 | 8693.0 | 16.53% | 8314.4 | 16.03% | motif file (matrix) | svg |
| 139 | A T C G G A C T C G A T C G T A C G T A C A G T G A T C G A T C G A T C A G C T | Otx2(Homeobox)/EpiLC-Otx2-ChIP-Seq(GSE56098)/Homer | 1e-2 | -6.899e+00 | 0.0032 | 6201.0 | 11.79% | 5892.8 | 11.36% | motif file (matrix) | svg |
| 140 | T A C G T A C G G T A C A T C G A C T G T A C G T C G A C T G A T C G A A T C G | E2F6(E2F)/Hela-E2F6-ChIP-Seq(GSE31477)/Homer | 1e-2 | -6.708e+00 | 0.0038 | 4933.0 | 9.38% | 4667.4 | 9.00% | motif file (matrix) | svg |
| 141 | T G A C C T A G A C T G T A G C C G A T A C T G A T G C C A T G A T C G A T C G A T C G T A G C C T G A T A G C G C T A A C T G C G T A A G C T C G T A C T G A | GATA:SCL(Zf,bHLH)/Ter119-SCL-ChIP-Seq(GSE18720)/Homer | 1e-2 | -6.685e+00 | 0.0039 | 679.0 | 1.29% | 595.2 | 1.15% | motif file (matrix) | svg |
| 142 | C T A G C T G A T C A G A T C G A G C T C A T G G A C T A G T C C T G A T G C A | Tbx6(T-box)/ESC-Tbx6-ChIP-Seq(GSE93524)/Homer | 1e-2 | -6.586e+00 | 0.0043 | 7360.0 | 14.00% | 7025.7 | 13.55% | motif file (matrix) | svg |
| 143 | C A T G A C G T A G T C G A T C G A T C G A T C G C T A C T A G C T A G C T A G T C A G T C G A | EBF1(EBF)/Near-E2A-ChIP-Seq(GSE21512)/Homer | 1e-2 | -6.570e+00 | 0.0043 | 9662.0 | 18.37% | 9268.1 | 17.87% | motif file (matrix) | svg |
| 144 | C T G A T C A G G C T A A G C T A G T C G A C T C T G A C T A G T G C A C T G A A G T C G T A C G A T C A C T G T C G A | ZBTB12(Zf)/HEK293-ZBTB12.GFP-ChIP-Seq(GSE58341)/Homer | 1e-2 | -6.529e+00 | 0.0045 | 2938.0 | 5.59% | 2745.7 | 5.29% | motif file (matrix) | svg |
| 145 | C A T G A C T G A G C T A T G C C G T A A T G C G T A C G A C T T A C G C T G A A C T G A C T G G C A T A T G C C T G A | THRb(NR)/HepG2-THRb.Flag-ChIP-Seq(Encode)/Homer | 1e-2 | -6.314e+00 | 0.0055 | 4125.0 | 7.84% | 3893.3 | 7.51% | motif file (matrix) | svg |
| 146 | C T G A A C G T A C G T A C G T A G T C G A C T C G A T C T G A A C T G C G T A C G T A T C G A | STAT5(Stat)/mCD4+-Stat5-ChIP-Seq(GSE12346)/Homer | 1e-2 | -6.291e+00 | 0.0056 | 2572.0 | 4.89% | 2397.3 | 4.62% | motif file (matrix) | svg |
| 147 | A G C T G C A T A C T G A C G T A G T C A C G T C T A G T A C G | Smad3(MAD)/NPC-Smad3-ChIP-Seq(GSE36673)/Homer | 1e-2 | -6.279e+00 | 0.0056 | 19426.0 | 36.94% | 18841.9 | 36.33% | motif file (matrix) | svg |
| 148 | C T A G A C T G T G C A G T C A A T G C C G T A A T C G A T G C A G T C C T A G | ZNF341(Zf)/EBV-ZNF341-ChIP-Seq(GSE113194)/Homer | 1e-2 | -6.199e+00 | 0.0060 | 4937.0 | 9.39% | 4681.2 | 9.03% | motif file (matrix) | svg |
| 149 | C G T A T A G C A G T C C T A G C A G T C T A G C T G A G T A C G C A T T C G A C G T A G C A T A G C T C T A G T C G A | PAX3:FKHR-fusion(Paired,Homeobox)/Rh4-PAX3:FKHR-ChIP-Seq(GSE19063)/Homer | 1e-2 | -6.120e+00 | 0.0065 | 1265.0 | 2.41% | 1151.6 | 2.22% | motif file (matrix) | svg |
| 150 | A C G T C T A G A G C T A C G T A C G T C T G A A G T C G A C T A G C T C G T A | FOXM1(Forkhead)/MCF7-FOXM1-ChIP-Seq(GSE72977)/Homer | 1e-2 | -6.049e+00 | 0.0069 | 7931.0 | 15.08% | 7594.3 | 14.64% | motif file (matrix) | svg |
| 151 | C T A G C A T G C A T G A G C T G A C T C G T A G A T C A G C T G T C A A T G C T C G A C T A G C A T G A C G T A G T C C T G A | LXRE(NR),DR4/RAW-LXRb.biotin-ChIP-Seq(GSE21512)/Homer | 1e-2 | -5.918e+00 | 0.0078 | 296.0 | 0.56% | 247.0 | 0.48% | motif file (matrix) | svg |
| 152 | C T G A A T G C G C T A C G A T A T G C C G T A C G T A C G T A C T A G T A C G | Tcf3(HMG)/mES-Tcf3-ChIP-Seq(GSE11724)/Homer | 1e-2 | -5.671e+00 | 0.0100 | 1729.0 | 3.29% | 1598.5 | 3.08% | motif file (matrix) | svg |
| 153 | C T G A T C A G A C T G C A G T A G T C G A C T A G T C A C G T C G T A G C T A A T G C G A T C | PRDM14(Zf)/H1-PRDM14-ChIP-Seq(GSE22767)/Homer | 1e-2 | -5.643e+00 | 0.0102 | 2362.0 | 4.49% | 2205.2 | 4.25% | motif file (matrix) | svg |
| 154 | C A T G G A C T T A C G G T C A G T A C G A T C G A C T A G C T A T C G T C G A T A C G T A G C | ERRg(NR)/Kidney-ESRRG-ChIP-Seq(GSE104905)/Homer | 1e-2 | -5.609e+00 | 0.0105 | 5986.0 | 11.38% | 5712.9 | 11.02% | motif file (matrix) | svg |
| 155 | C A T G A G C T T A C G G T C A G T A C T A G C A G C T G A C T A T C G T C G A | Esrrb(NR)/mES-Esrrb-ChIP-Seq(GSE11431)/Homer | 1e-2 | -5.415e+00 | 0.0126 | 4722.0 | 8.98% | 4490.0 | 8.66% | motif file (matrix) | svg |
| 156 | C T A G C A T G C A T G T A C G A G T C G C A T A G C T C T A G A C G T A G T C G A C T A C T G A C T G A C T G T C G A | Zfp809(Zf)/ES-Zfp809-ChIP-Seq(GSE70799)/Homer | 1e-2 | -5.373e+00 | 0.0131 | 2136.0 | 4.06% | 1992.9 | 3.84% | motif file (matrix) | svg |
| 157 | C T A G C T A G A C T G A G C T G A C T A G C T A C G T C T A G G T C A C T G A A C T G T C A G C T G A A C G T C T A G G C T A C T G A A C G T C G T A C T A G A C T G C G T A A C T G A G C T A C G T | ZFP3(Zf)/HEK293-ZFP3.GFP-ChIP-Seq(GSE58341)/Homer | 1e-2 | -5.316e+00 | 0.0138 | 28.0 | 0.05% | 16.4 | 0.03% | motif file (matrix) | svg |
| 158 | G C T A T C G A C G T A C T A G A G C T G T C A G T C A C G T A A G T C C G T A | FOXA1(Forkhead)/LNCAP-FOXA1-ChIP-Seq(GSE27824)/Homer | 1e-2 | -4.961e+00 | 0.0195 | 9132.0 | 17.37% | 8796.1 | 16.96% | motif file (matrix) | svg |
| 159 | C G T A C T G A C G T A C T A G T C G A C T A G A C T G C G T A C G T A T A C G A G C T A T C G | SpiB(ETS)/OCILY3-SPIB-ChIP-Seq(GSE56857)/Homer | 1e-2 | -4.823e+00 | 0.0222 | 1667.0 | 3.17% | 1550.9 | 2.99% | motif file (matrix) | svg |
| 160 | C A G T A C T G C G T A G T C A C G T A A G C T G A T C A G C T A C T G C G T A A G T C G T C A A G T C A G T C A T C G | Six4(Homeobox)/MCF7-SIX4-ChIP-Seq(Encode)/Homer | 1e-2 | -4.739e+00 | 0.0240 | 311.0 | 0.59% | 267.3 | 0.52% | motif file (matrix) | svg |
| 161 | C G A T C T A G C T G A A T G C C T G A T C G A C G T A C T G A T C G A T A G C A G T C C G T A A C T G T C G A A T G C | Hand2(bHLH)/Mesoderm-Hand2-ChIP-Seq(GSE61475)/Homer | 1e-2 | -4.682e+00 | 0.0253 | 3565.0 | 6.78% | 3383.2 | 6.52% | motif file (matrix) | svg |
